# A Mobile Sleep-Management Learning System for Improving Students’ Sleeping Habits by Integrating a Self-Regulated Learning Strategy: Randomized Controlled Trial

**DOI:** 10.2196/11557

**Published:** 2018-10-29

**Authors:** Hui-Chun Chu, Yi-Meng Liu, Fan-Ray Kuo

**Affiliations:** 1 Department of Computer Science and Information Management School of Business Soochow University Taipei Taiwan

**Keywords:** cognitive behavioral therapy, insomnia, mobile phone, self-regulated learning

## Abstract

**Background:**

Insomnia can significantly affect students’ learning performance. Researchers have indicated the importance and challenge of coping with insomnia using nondrug treatments, such as cognitive behavioral therapy (CBT) for insomnia. However, it is easy for the traditional CBT for insomnia to be interrupted owing to the overly lengthy period of sleep therapy. Self-regulated learning (SRL) strategies are known to be an effective approach for helping students improve their time management, as well as their ability to set learning goals and adopt learning strategies.

**Objective:**

The objective of this study was to propose a mobile sleep-management learning system integrated with SRL strategies and CBT.

**Methods:**

A total of 18 undergraduate students from a university in northern Taiwan participated in the 2-week experiment of using this sleep-management system.

**Results:**

The experimental results showed that the proposed approach was useful and easy for students to use. In addition, the number of students with insomnia significantly decreased; that is, the proposed approach could help students improve their sleep quality and cultivate better sleeping habits, which is important for them to enhance their learning efficiency.

**Conclusions:**

With the assistance of this proposed approach, students can plan their daily life by setting goals, applying strategies, monitoring their life habits process, and modifying strategies to cultivate good learning and healthy lifestyle habits.

**Trial Registration:**

Government Research Bulletin MOST104-3011-E038-001; https://www.grb.gov.tw/search/planDetail? id=11568383&docId=467988 (Archived by WebCite at http://www.webcitation.org/73MnPHNri)

## Introduction

### Background

Sleep problems, especially insomnia, affect many people in their daily life. It is found that >25% of adults experience insomnia [1]. They also indicated that insomnia has a negative impact on both health and psychological well-being. Researchers argued that sleep problems such as sleep disturbances are highly associated with several issues such as psychological demands, low recognition, low social support, perception of danger, emotional demands, and work-life imbalance [2]. Moreover, some studies indicated that unhealthy lifestyles and habits (eg, smoking) are associated with increased risk of sleep problems [3]. For example, Shimura et al. showed that caffeine intake at night, using electronic displays within 2 hours of sleep, and irregular timing of meals may be associated with sleep problems such as sleep disturbance and daytime sleepiness [4]. In the past decades, numerous studies have investigated sleep medical care issues, and a large portion of these studies have recommended nondrug treatment, in particular, CBT for insomnia [5,6]. Researchers have also indicated that insomnia is usually caused by poor time management and could significantly affect students’ learning performance [7-9].

**Figure 1:**
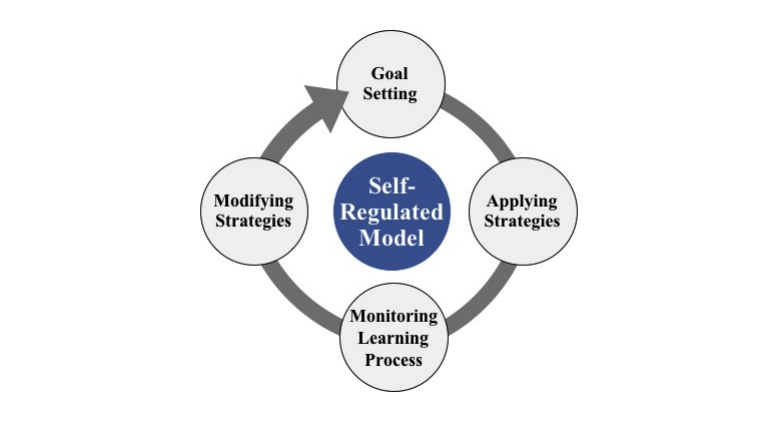
The self-regulatory learning cycle.

Self-regulated learning (SRL) is known to be an effective way to help students manage their learning plans [10-12]. With the help of the SRL approach, students are guided to plan their own learning goals and time management as strategies to improve their learning outcomes; moreover, they are guided to periodically make reflections and modify their plans [13,14]. Zimmerman et al proposed a self-regulatory learning cycle consisting of 4 stages, as shown in Figure 1 [15]. In the learning field, students need to do “goal setting” in the first SRL stage. They are asked to analyze their learning process and set their goals based on the proposed learning strategies. They usually set goals based on their prior experience, such as the degree of completion of reading learning materials, average time to learn, and the choices of learning environments such as learning with their peers, so that they could learn at their own pace by setting a series of learning goals. The second stage is “applying proper strategies.” Students need to carry out the learning plan that was outlined in the goal-setting stage to complete their learning tasks by using the chosen learning strategies such as “asking questions online” and “making annotations.” The third stage is “monitoring the learning process.” Students realize what they have done and the effectiveness of the strategies depending on whether their performance reaches the goals they have set. The last stage is “modifying strategies.” In this stage, students would evaluate whether the goals they set in the goal-setting stage are suitable for their learning performance. In addition, they evaluate the completion of their goals. Therefore, they could modify their strategies to suit them better to cultivate adequate sleeping habits.

Researchers have indicated that the self-regulated model can help students regulate themselves and help them have better learning habits [14]. In addition, Shimura et al addressed that each type of sleep problem has its own associated life habit factors [4]. Unhealthy lifestyle or habits (eg, playing with mobile electronic devices before sleep time) may easily lead to sleep problems. Therefore, to help students cultivate a good lifestyle, healthy habits, and readiness for higher quality sleep, a mobile sleep-management learning system, the sleep-management system based on the self-regulated learning strategy (SMSR) is proposed in this study; it is designed on the basis of 4 stages of the SRL strategy proposed [15].

The application of smartphones to deliver health-related content has experienced rapid growth in recent years, with >325,000 mobile health (mHealth) apps currently available in the digital marketplace [16]. However, most of these apps play the role of delivering accessible health-related knowledge and support to users. The effectiveness of an mHealth app usually depends on users’ initiative. When it comes to effective data logging and feedback to users, researchers showed that using a mobile app with a wearable device as a nondrug treatment to treat insomnia could effectively reduce the number of sleeping pills taken [17]. Researchers mentioned that showing the discrepancy between recorded data and sleep diaries is more effective than verbal feedback when users monitor themselves [18]; that is, it is necessary to include both score feedback and diary review functions to guide users to double check their conditions. It is, therefore, important to propose an effective approach integrated with wearable devices to assist students in fostering good daily life habits and managing their time.

After developing the proposed approach, an experiment was conducted to evaluate the effectiveness of the mobile system with the following questions:

- Is the SMSR mobile learning system useful and easy for users to use?
- Can the SMSR mobile learning system help users to improve their sleep quality and manage their sleep habits actively?
- Can the SMSR mobile learning system that provides indicators and feedback based on the rule-based expert system help users to keep a diary?

### Literature Review

#### Sleep Quality Issues

Sleep problems include difficulty falling asleep and low sleep quality such as insomnia, sleep disturbances, and sleep disorders. Researchers indicated that insomnia negatively impacts both health and psychological well-being [1]. Specifically, sleep problems may also increase the risk of morbidity and mortality [19]. Various studies have found that sleep problems affect the quality of life and daytime energy, and lead to anxiety and depression [1,2,19-21]. In addition, researchers have indicated that sleep problems could be highly associated with several issues such as psychological demands, low recognition, low social support, perception of danger, emotional demands, and work-life imbalance [2], and could influence both older and younger people [19].

Several studies have found that students often suffer from lack of sleep [9,22-24]; these studies also reavealed that suffering from poor sleep quality could greatly impact students’ daily life, learning performance, motivation, self-efficacy, and so on. Moreover, some researchers pointed out that students with sleep problems tend to have a low intention to seek help [24].

In the past decades, many studies have investigated sleep medical care issues, and pharmacotherapy is the most frequently used intervention for insomnia [25]. A large portion of these studies have also recommended nondrug treatment; in particular, CBT for insomnia is effective for improving the sleep quality [5,6]. In addition, a study conducted with 3 groups, CBT, pharmacotherapy, and combination therapy, found that the CBT alone had a greater long-term effect on sleep latency and sleep efficiency than the combined treatment group [25]. Researchers argued that the sleep quality of university students is usually impaired because of high academic stress and time management problems [22]. Therefore, improving the sleep quality through CBT, time management, and sleep-related personal traits is a critical issue.

#### Mobile Technologies for Enhancing Health

With the rapid development of technology, smart devices, such as smartphones, tablets, and all kinds of wearable devices, have become popular all over the world [26]. At the same time, mHealth apps have stepped in [27]. Mobile devices could help with data collection, care delivery, patient communication, and real-time monitoring of health conditions [28]. In addition, mHealth apps have been used as interventions for various diseases. For example, Seiler et al [29] reviewed the treatment of fatigued cancer survivors and found that electronic health interventions appeared to be effective for managing their fatigue. Researchers have also predicted that mHealth and electronic health interventions would play critical roles in future personal care delivery [29]. Moreover, researchers carried out clinical evaluation of an mHealth app for stress management and found that patients who used the mobile classifier and an associated mHealth app were more willing to continue the treatment and significantly improved on measures of stress, anxiety, and anger compared with those who controlled with CBT alone [30].

When it comes to the effective collection of data logs and feedback to users, researchers showed that using a mobile app with a wearable device as a nondrug treatment to treat insomnia could effectively reduce the number of sleeping pills taken [17]. Researchers mentioned that showing the discrepancy between recorded data and sleep diaries is more effective than verbal feedback when users monitor themselves [18]; that is, it is necessary to include both score feedback and diary review functions to guide users to double check their condition. It is, therefore, important to propose an effective approach integrated with wearable devices to assist students in fostering good daily life habits and managing their time. mHealth approaches integrated with mobile apps and wearable device sensors could potentially provide treatment and personal care at a low cost [30]. In their study, mobile apps were used to measure the users’ health condition and collected data logs and feedback from users.

## Methods

### The Design of a Mobile Sleep-Management System Integrating the Self-Regulated Learning Strategy

**Figure 2:**
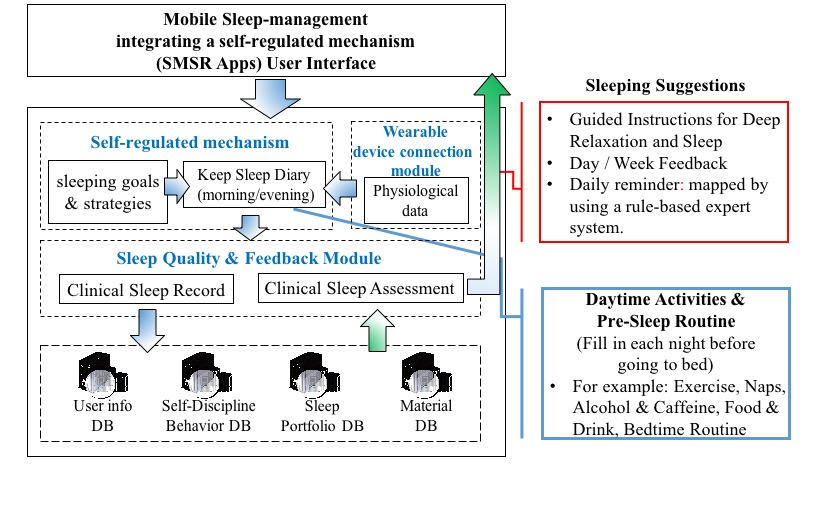
The sleep-management system based on the self-regulated learning strategy (SMSR) structure. DB: databases.

In this study, a mobile sleep-management system integrating an SRL strategy was developed for use on Android mobile devices. Figure 2 shows the structure of the system. The *self-regulated mechanism* consists of setting sleeping goals and strategies and keeping a sleep diary for morning and evening activities. This module was designed for users to provide their daily behavior information and set their sleep goals. The *wearable device connection module* providing physiological data could help users to keep a diary based on the physiological data provided. The *sleep quality and feedback module* consists of clinical sleep records and clinical sleep assessment. The clinical sleep records are uploaded to the following databases: the user information database containing background information, the self-discipline behavior database containing users’ self-regulated behavior data, the sleep portfolio database containing sleep behavior data, and the material database providing health educational materials for users. The clinical sleep assessment provides users with sleeping suggestions, such as guided instructions, for sleep, daily reminders, and daily and weekly feedback.

**Figure 3:**
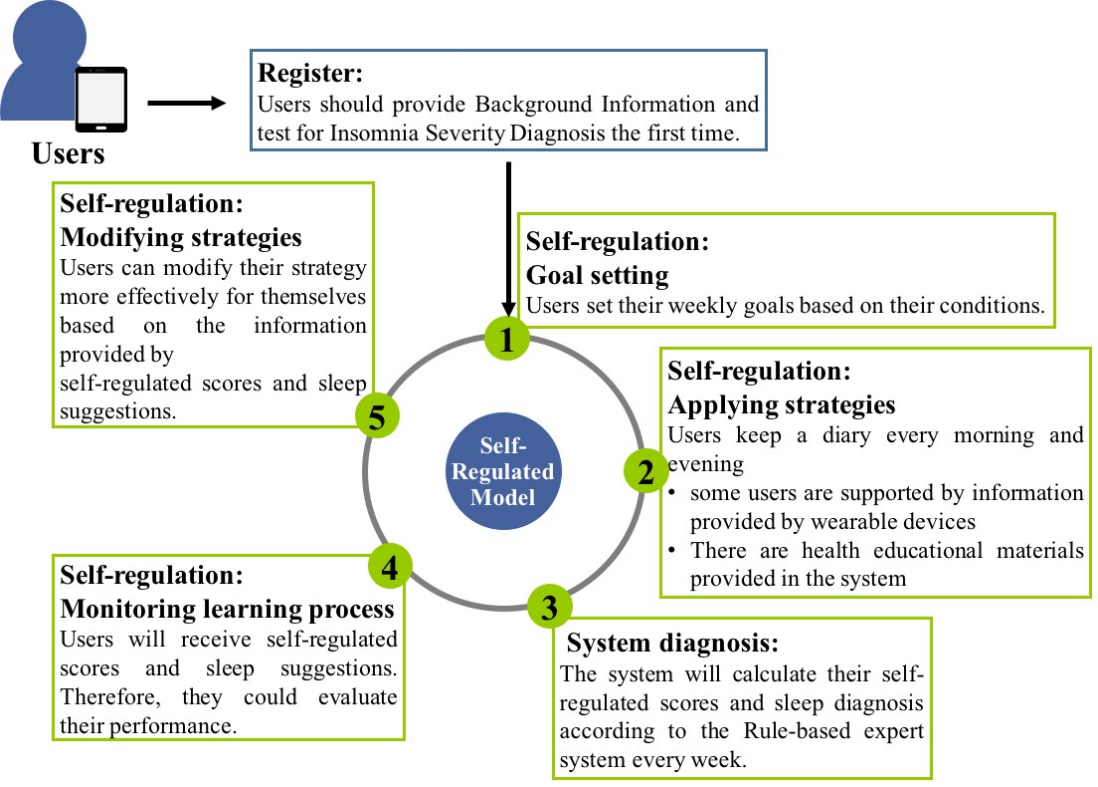
The mobile sleep-management system procedure.

Figure 3 shows the procedure of using the mobile sleep-management system. Users register to use the SMSR and answer several questions for insomnia severity diagnosis. After the initial sleep and health habits evaluation, users will start using the self-regulated model based on their individual condition. The first step is the goal-setting stage; the SMSR lets users set goals for 5 items, namely sleep, exercise, relaxation activities, drinking and eating (including caffeine drinks), and sleep environment. In the second stage, users can set up the strategies they want to follow in the next week. The SMSR system will ask users to keep a diary every day of their activities in the morning and evening for applying their strategies.

In the third stage, the monitoring stage, users keep a diary to meet the goals they set. The SMSR will calculate their self-regulated scores and sleep diagnosis according to the rule-based expert system and their weekly system log. The system then provides the sleep scores to show users’ completion of their goals.

In the fourth stage, modifying strategies, users will receive self-regulated scores and sleep suggestions. The SMSR provides sleep suggestions that could diagnose users’ sleep quality and lifestyle habits. The information provided will help users in the next-stage goal setting.

By using this SMSR lifestyle strategy, users could see the evaluation of their lifestyle self-regulated performance. At the end of the week, users can modify their strategy more effectively based on the self-regulated scores and sleep suggestions. The SMSR cycle lasts for 7 days, with users receiving feedback from the system every Sunday. After that, they should modify their strategies and set goals for the following week.

**Figure 4:**
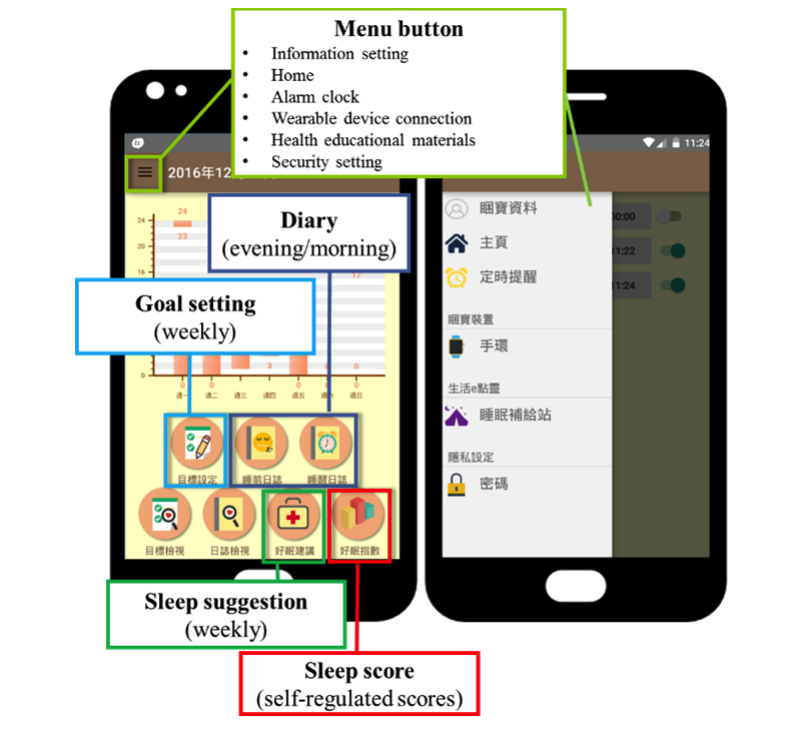
The home screen of the sleep-management system based on the self-regulated learning strategy mobile sleep-management system.

Figure 4 shows the main function on the home screen of the SMSR, including goal setting, diary, sleep score, sleep suggestions, and menu buttons.

**Figure 5:**
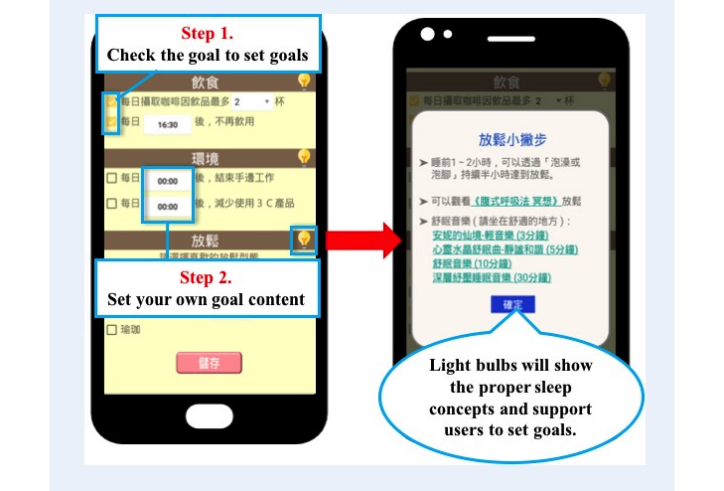
The goal-setting stage interface.

Figure 5 shows the interface of the goal-setting function. The goals are divided into 5 items, namely, sleep goals, exercise goals, drink and food goals, sleep environment goals, and relaxation activity goals. Users check the goals they want to set, and then fill in the specific content. For example, a user could set a sleep goal and fill in the time he/she will go to sleep by, such as 10 pm. In addition, the goal setting has a light bulb button to help users get proper concepts to support their goal-setting process (Figure 5). For instance, sleep tips will be given such as “taking a bath for half an hour or light music would make your mind relax before sleep.”

**Figure 6:**
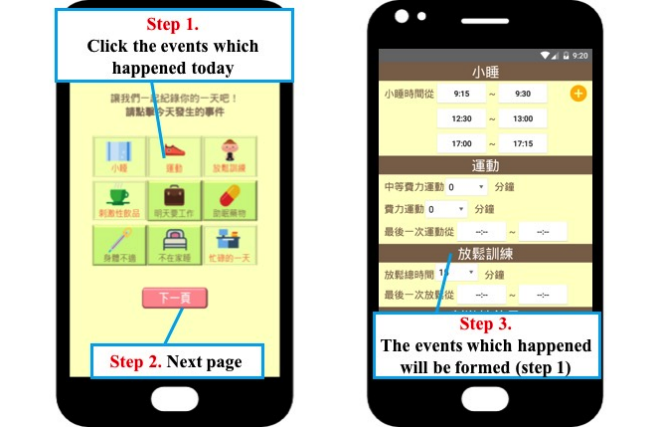
The diary recording interface based on cognitive behavioral therapy for insomnia.

Figure 6 shows the evening diary interface. Users can click the events that happened during the day and click the button to go to the next page. The diary will be formed according to the events the users clicked, meaning that they can complete the process easily and that the complexity is reduced.

**Figure 7:**
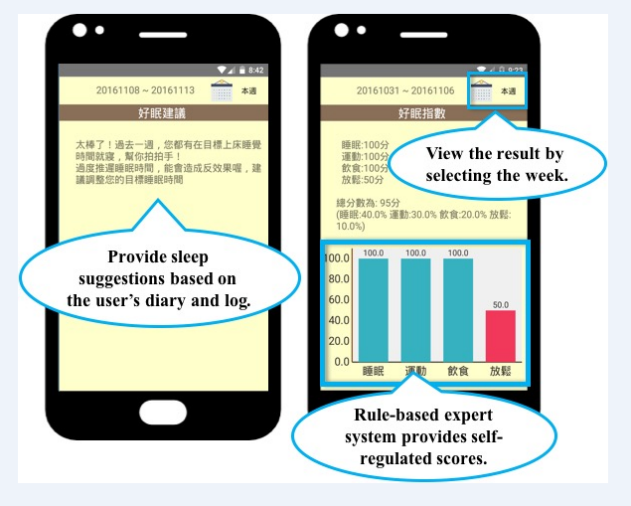
The feedback interface.

Figure 7 shows the feedback interface. Users receive the information every week to learn about their self-regulated performance and sleep suggestions. Figure 7 shows the self-regulated scores calculated by the expert system. Users can have scores for the goal items they set. They can monitor themselves by the feedback of the scores. Figure 7 also shows that users are allowed to modify their strategies based on the sleep suggestions provided by the rule-based expert system.

### Experimental Design

#### Participants

In this study, participants were undergraduate students from a university in northern Taiwan; there were 18 students, including 3 males and 15 females. Among them, 1 was diagnosed with severe insomnia, 4 with moderate insomnia, and 12 with subthreshold insomnia. Most of them had had experience of sleep disturbance; some of them also mentioned that they had suffered from sleep problems for >3 months. Of 18 participants, 10 used the SMSR with wearable devices, while the other 8 used the SMSR without wearable devices. The participants’ average age was 22 years: mean 22 (SD 1.286) years.

#### Experimental Procedure

**Figure 8:**
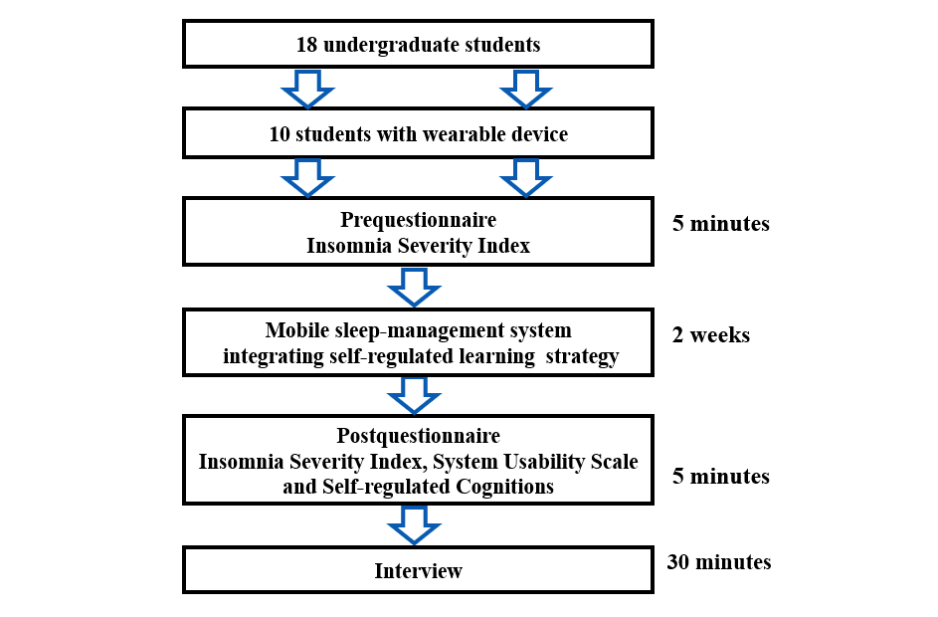
The experimental procedure.

Figure 8 shows the experimental procedure. Before students started using the SMSR mobile sleep-management system, they were required to complete the prequestionnaire Insomnia Severity Index (ISI) to identify their sleep conditions. After that, they were asked to use the mobile sleep-management system for 2 weeks. After the 2 weeks, they completed 3 postquestionnaires, the ISI, the content of which was the same as the prequestionnaire, the System Usability Scale (SUS), and the self-regulated cognitions questionnaire. We asked students to complete the ISI for both the pre- and postquestionnaire so that we could compare them to find whether using the mobile sleep-management system could help them improve their sleep quality by self-regulation. Finally, we interviewed students about using the SMSR.

#### Measuring Tools

The postquestionnaires used in this study were the ISI, SUS, and self-regulated cognition questionnaire. The ISI consists of 5 items with a 5-point Likert scale. The first item is about the current severity of insomnia problems, including difficulty falling asleep, difficulty staying asleep, and problems waking up too early, on a scale of 0 (*None*) to 4 (*Very Severe*). The second item is about satisfaction with the current sleep pattern on a scale of 0 (*Not At All Noticeable*) to 4 (*Very Much Noticeable*). The third item is the degree to which sleep problems are noticeable to others on a scale of 0 (*Not At All Worried*) to 4 (*Very Much Worried*). The fourth item is about worry over your current sleep pattern on a scale of 0 (*Not At All Worried*) to 4 (*Very Much Worried*). The last item is interference of current sleep on a scale of 0 (*Not At All Interfering*) to 4 (*Very Much Interfering*). The total score could sort respondents into 4 groups according to their insomnia severity as follows: absence of insomnia (score range: 0-7); subthreshold insomnia (score range: 8-14); moderate insomnia (score range: 15-21); and severe insomnia (score range: 22-28).

We summed the scores and measured the degree of insomnia, where the higher the score, the more serious the problem [31]. Reportedly, the ISI was used to measure insomnia and found to have a Cronbach alpha of 0.74 [31]; in this study, it reached a Cronbach alpha of 0.77.

The SUS and the questionnaire of self-regulated cognition were developed by 1 experienced teacher and 4 assistants who had used a mobile sleep-management system and wearable devices. The SUS has 4 items. The first is about whether the app is a good way to record daily behavior. The second is about whether the app helps them to reach their goals. The third is whether the users mind if they reach their goals when filling in the diary. The last item is about whether wearable devices help users to fill in the diary faster. The Cronbach alpha was 0.76. Finally, the questionnaire of self-regulated cognition has 4 items about whether the SMSR functions help users with self-regulation. The Cronbach alpha value was 0.88.

The interviews focused on whether the mobile sleep- management system could help build proper sleep concepts and knowledge and improve the users’ sleep quality. In addition, we asked those students who used a wearable device with the SMSR whether it improved their motivation, and whether the support system improved their self-regulation.

## Results

In this study, a paired *t* test was used to investigate the differences between the pre- and postquestionnaires of the ISI to identify whether there was any improvement in their sleep quality (Table 1). For scale 1 items, “*Difficulty falling asleep*,” a significant difference was found (*P*=.04), indicating a decrease in the difficulty of falling asleep for participants. For scale 5, “*To what extent do you consider your sleep problems to INTERFERE with your daily functioning (e.g., mood) CURRENTLY?*”, there was a significant difference (*P*=.002); that is, the interference of sleep problems decreased. Scale 6, “*How NOTICEABLE to others do you think your sleep problems are in terms of impairing the quality of your life?*” shows that the mean of the scores decreased when comparing the pre- and postquestionnaires, indicating that participants’ sleep problems reduced (*P*=.005). For scale 7, “*How WORRIED/DISTRESSED are you about your current sleep problems?*,” significant differences showed that the worry about sleep problems reduced (*P*=.02). The mean of the total scores decreased to 14.94 from 18.50; that is, the SMSR was successful in reducing the severity of participants’ insomnia, and their sleep quality improved as well. Table 2 shows the percentage of people divided into 4 groups according to the degree of insomnia severity. After using the SMSR system, the numbers in the absence of insomnia and subthreshold insomnia groups increased, whereas the numbers in the moderate insomnia and severe insomnia groups decreased.

Table 3 shows the statistics of the SUS. It was found that users thought that using the SMSR could help them keep a diary, and the interface of the SMSR was user-friendly and easy to use, with means of 3.78 and 3.89. In addition, users showed a positive attitude toward wearable devices when using it with the SMSR system, with means of 3.69 and 3.75, indicating that wearable devices could enhance their motivation and provide information about sleep routines to help them know more about their sleeping habits.

After the postquestionnaire for the system usability, students were asked about the usability of the system. Most expressed positive comments on the ease of use and friendly interface of the SMSR system as in the following example:

The system interface is very friendly and the provision of system logs is helpful to me for tracing my daily situation. These facilities enable me to understand time management and my sleep routine.

**Table 1:** Paired *t* test for the pre- and postquestionnaires of the Insomnia Severity Index.

Items	Pre, mean (SD)	Post, mean (SD)	*t*
Scale 1. Please rate the CURRENT (ie, LAST 2 WEEKS) SEVERITY of your insomnia problem(s). (Q1. Difficulty falling asleep)	2.33 (0.84)	1.78 (0.55)	*t*_17_=2.26^a^
Scale 5. To what extent do you consider your sleep problems to INTERFERE with your daily functioning (e.g., mood) CURRENTLY?	3.50 (0.62)	2.61 (0.98)	*t*_17_=3.69^b^
Scale 6. How NOTICEABLE to others do you think your sleep problems are in terms of impairing the quality of your life?	2.83 (1.15)	2.00 (1.19)	*t*_17_=3.22^b^
Scale 7. How WORRIED/DISTRESSED are you about your current sleep problems?	3.11 (0.96)	2.33 (1.08)	*t*_17_=2.61^a^

^a^*p*<.05.

^b^*p*<.01.

**Table 2:** Paired *t* test for the pre- and postquestionnaires of the Insomnia Severity Index.

Level of insomnia	Before experiment (%)	After using SMSR^a^ (%)	Difference (%)
Absence of insomnia (total score: 0-7)	0	5.5	>5.5
Subthreshold insomnia (total score: 8-14)	5.5	55.6	>50.1
Moderate insomnia (total score: 15-21)	77.8	27.8	−50
Severe insomnia (total score: 22-28)	16.7	11.1	−5.6

^a^SMSR: sleep-management system based on the self-regulated learning strategy.

**Table 3:** Statistics of the System Usability Scale (n=18).

Items	Mean (SD)
Scale 1: I think it’s quite nice to log my daily diary using the SMSR.	3.78 (0.65)
Scale 2: I think the SMSR interface is user-friendly and easy to use.	3.89 (0.96)
Scale 3: Wearable devices let me know more about my sleep routine.	3.69 (1.03)
Scale 4: Wearable devices can motivate me to modify my sleep routine.	3.75 (1.14)

^a^SMSR: sleep-management system based on the self-regulated learning strategy.

**Table 4:** The statistics of self-regulated cognition (n=18).

Scale and items		Mean (SD)
**Scale 1. Goal setting**		
	Q1. Setting the goals (eg, sleep goals, exercise goals) could make me pay more attention to my daily routine.	3.89 (0.32)
	Q2. It could make me modify my daily routine according to the goals (eg, sleep goals, exercise goals).	3.94 (0.64)
	Q3. The light bulb hint could help me know proper life routines.	3.56 (0.62)
**Scale 2. Applying strategies**		
	Q1 The evening/morning diaries make me notice my life routine.	4.00 (0.69)
	Q2 The evening/morning diaries force me to reach my goals.	3.78 (0.65)
	Q3 I will be more attentive to whether I reach the goals when keeping a diary.	4.00 (0.69)
	Q4 Wearable devices could help me keep the diary.	3.83 (1.19)
**Scale 3. Monitoring process**		
	Q1 The sleep scores make me notice more about my life routine.	3.78 (1.11)
	Q2 Sleep scores can help me modify my life routine.	3.50 (1.10)
	Q3 Sleep scores can help me improve my sleep quality.	3.44 (0.98)
**Scale 4. Modifying strategies**		
	Q1 Sleep suggestions could make me notice my life routine.	3.83 (1.15)
	Q2 Sleep suggestions could let me know my status of reaching goals.	3.78 (1.06)
	Q3 Sleep suggestions could help me modify my strategies.	3.78 (1.00)

Table 4 presents the statistical results for the questionnaire of self-regulated cognition. For scale 1, “*goal setting*,” the mean scores were >3.5, showing that most users agreed with keeping a diary by the SMSR. For scale 2, “*Applying strategies*,” the items of “*The evening/morning diaries make me notice my life routine*” and “*I will be more attentive to whether I reach my goals when keeping a diary*” have mean scores of 4, indicating that the SMSR could help users notice their life routine and whether they reach their goals. Every mean score in scale 3 was >3, indicating that sleep scores could help users to monitor themselves. For scale 4, “*modifying strategies*” shows that every mean score was >3.5; that is, sleep suggestions help modify the strategies for the coming week. For the self-regulated cognition, students expressed their ideas about the self-regulated strategy embedded in the CBT in the interview process, with one respondent stating:

The system could guide me to achieve better sleeping quality step-by-step by means of the self-regulated strategy. I think it is quite an important factor to manage our own sleeping behaviors and monitor time management ourselves, which is a potential way to enhance sleep-related traits.

Specifically, 10 students who used the SMSR with wearable devices were surveyed to identify the extent to which the wearable devices facilitated the execution of the plan. There were totally 3 questions for students, including 2 items for the usability of the devices, and 1 for the diary record, as shown in Table 5. Each mean score for the 3 items was >4.0, showing that most of them agreed with the usability of SMSR with a wearable device for recording their sleep routine and keeping a diary.

For the usability of a wearable device, most of them expressed positive comments on the functions of the SMSR system with the wearable device, as below:

The wearable device enables me to know and adjust my sleep routine easily. That really helps me pay attention to my sleep situation.

**Table 5:** The statistics of the usability of the sleep-management system based on the self-regulated learning strategy (SMSR) with wearable devices (n=10).

Items	Mean (SD)
Q1. Wearable devices let me know more about my sleep routine.	4.00 (0.67)
Q2. Wearable devices provide motivation for me to modify my sleep routine.	4.10 (0.74)
Q3. Wearable devices could help me keep the diary.	4.20 (0.79)

## Discussion

### Prinicpal Findings

In this study, a mobile sleep-management system integrating SRL strategies, named the SMSR, was designed and tested; it was designed on the basis of most well-known CBT for insomnia and the effective learning strategy, SRL [15,32].

From the experimental results, it was found that the proposed systems effectively reduced students’ worry about their sleep problems. To sum up, the SMSR mobile system is easy to use and useful to users according to the SUS. It seems that users are satisfied with the SMSR interface and think it is easy to use. Moreover, it could help users to improve their sleep quality and manage their sleeping habits on their own by providing them with sleep scores and sleep suggestions, as indicated elsewhere [18]. The well-designed feedback in the mHealth app could help users monitor themselves by providing logging information. According to the findings, the SMSR helps the most in the applying strategies phase; it could also provide indicators and feedback based on the rule-based expert system to help users keep a diary. The researchers found that those who used mobile apps and stress algorithm were more likely to complete their goals and demonstrated reduced stress and anger compared with the control group. It can be inferred from the results that the SMSR integrated with CBT and the SRL strategy helped enhance users’ cognition of sleep hygiene; this finding is consistent with that reported in another study [33], showing that CBT integrated with mobile apps would be a positive treatment for patients with insomnia. On the other hand, this study also found that users could gain benefits by using the SRL strategy integrated with a mobile app, no matter whether they were using wearable devices or not; this implies that the guidance provided in the self-monitoring process could help users be aware of their own situations and, hence, improve their sleep quality. Therefore, it is suggested that researchers not only need to develop innovative technologies but also need to focus on how to cultivate patients’ living habits by using efficient autonomy-learning strategies.

Researchers also pointed out that mHealth approaches integrated with mobile apps and wearable device sensors could potentially provide treatment and personal care at a low cost [30]. The SMSR integrated with CBT, and the SRL strategy could help students manage their sleep time, monitor the extent to which their goals are achieved, and modify their strategies accordingly for the coming week; this finding is consistent with that of another study [22], indicating that university students may significantly improve their sleep quality and sleep-related personality traits. Furthermore, some studies reported similar findings, showing that the use of mobile devices could improve users’ management of their sleep hygiene and quality [34].

### Conclusions

It is found that few students seek help for sleep problems. In this study, the SMSR may help students reduce their sleep problems by self-regulation [24]. Based on the preliminary findings of this pilot study, we will increase the number of the targeted population and keep collecting more qualitative and quantitative evidence for the proposed model regarding the sleep quality of university students. Moreover, researchers aimed to predict the sleep quality by analyzing the data collected by a wearable medical device using deep learning [35]. It may be an important issue in the future to integrate deep learning techniques into this study to analyze users’ behavior patterns and analyze how users reach or lose the goal for improving their sleep quality. It is expected that the proposed model could be applied in clinical settings for patients with insomnia.

## Appendix

Multimedia Appendix 1 CONSORT-EHEALTH checklist (V 1.6.1).

## Abbreviations

CBT: cognitive behavioral therapy
ISI: Insomnia Severity Index
SMSR: sleep-management system based on the self-regulated learning strategy
SRL: self-regulated learning
SUS: System Usability Scale

## References

[1] Kao C, Huang C, Wang M, Tsai P (2008). Insomnia: prevalence and its impact on excessive daytime sleepiness and psychological well-being in the adult Taiwanese population. Qual Life Res.

[2] Chazelle E, Chastang J, Niedhammer I (2016). Psychosocial work factors and sleep problems: findings from the French national SIP survey. Int Arch Occup Environ Health.

[3] Kanerva N, Pietiläinen O, Lallukka T, Rahkonen O, Lahti J (2018). Unhealthy lifestyle and sleep problems as risk factors for increased direct employers' cost of short-term sickness absence. Scand J Work Environ Health.

[4] Shimura A, Hideo S, Takaesu Y, Nomura R, Komada Y, Inoue T (2018). Comprehensive assessment of the impact of life habits on sleep disturbance, chronotype, and daytime sleepiness among high-school students. Sleep Med.

[5] Jungquist CR, O'Brien C, Matteson-Rusby S, Smith MT, Pigeon WR, Xia Y, Lu N, Perlis ML (2010). The efficacy of cognitive-behavioral therapy for insomnia in patients with chronic pain. Sleep Med.

[6] Savard J, Simard S, Ivers H, Morin CM (2005). Randomized study on the efficacy of cognitive-behavioral therapy for insomnia secondary to breast cancer, part II: Immunologic effects. J Clin Oncol.

[7] Baker FC, de Zambotti M, Colrain IM, Bei B (2018). Sleep problems during the menopausal transition: prevalence, impact, and management challenges. Nat Sci Sleep.

[8] Fortier-Brochu E, Beaulieu-Bonneau S, Ivers H, Morin CM (2012). Insomnia and daytime cognitive performance: a meta-analysis. Sleep Med Rev.

[9] Titova OE, Hogenkamp PS, Jacobsson JA, Feldman I, Schiöth HB, Benedict C (2015). Associations of self-reported sleep disturbance and duration with academic failure in community-dwelling Swedish adolescents: sleep and academic performance at school. Sleep Med.

[10] Michalsky T, Schechter C (2013). Preservice teachers' capacity to teach self-regulated learning: Integrating learning from problems and learning from successes. Teaching and Teacher Education.

[11] Subramanian R (2003). Resource ReviewsOrganizational and Educational Change: The Life and Role of a Change Agent Group, edited by BartunekJean M. Mahwah, NJ: Lawrence Erlbaum Associates, Inc., 2003. 295 pages, hard cover. AMLE.

[12] Siadaty M, Gašević D, Jovanović J, Pata K, Milikić N, Holocher-Ertl T, Jeremić Z, Ali L, Giljanović A, Hatala M (2012). Self-regulated Workplace Learning: A Pedagogical Framework and Semantic Web-based Environment. Journal of Educational Technology & Society.

[13] Azevedo R, Cromley JG (2004). Does Training on Self-Regulated Learning Facilitate Students' Learning With Hypermedia?. Journal of Educational Psychology.

[14] Tabuenca B, Kalz M, Drachsler H, Specht M (2015). Time will tell: The role of mobile learning analytics in self-regulated learning. Computers & Education.

[15] Zimmerman B, Bonner S, Kovach R (1996). Developing self-regulated learners: Beyond achievement to self-efficacy. American Psychological Association.

[16] research2guidance (2017). Abstract retrieved July 10,, from.

[17] Chen Y, Hung Y, Chen H (2016). Mobile Application-Assisted Cognitive Behavioral Therapy for Insomnia in an Older Adult. Telemed J E Health.

[18] Tang NKY, Harvey AG (2006). Altering misperception of sleep in insomnia: behavioral experiment versus verbal feedback. J Consult Clin Psychol.

[19] Crowley K (2011). Sleep and sleep disorders in older adults. Neuropsychol Rev.

[20] Alvaro PK, Roberts RM, Harris JK (2013). A Systematic Review Assessing Bidirectionality between Sleep Disturbances, Anxiety, and Depression. Sleep.

[21] Parish JM (2009). Sleep-related problems in common medical conditions. Chest.

[22] Schlarb AA, Friedrich A, Claßen M (2017). Sleep problems in university students - an intervention. Neuropsychiatr Dis Treat.

[23] Whitaker BN, Fisher PL, Jambhekar S, Com G, Razzaq S, Thompson JE, Nick TG, Ward WL (2018). Impact of Degree of Obesity on Sleep, Quality of Life, and Depression in Youth. J Pediatr Health Care.

[24] Zochil ML, Thorsteinsson EB (2017). Exploring poor sleep, mental health, and help-seeking intention in university students. Aust J Psychol.

[25] Jacobs GD, Pace-Schott EF, Stickgold R, Otto MW (2004). Cognitive behavior therapy and pharmacotherapy for insomnia: a randomized controlled trial and direct comparison. Arch Intern Med.

[26] Jonassen DH, Carr C, Yueh H (1998). Computers as mindtools for engaging learners in critical thinking. TECHTRENDS TECH TRENDS.

[27] Becker S, Miron-Shatz T, Schumacher N, Krocza J, Diamantidis C, Albrecht U (2014). mHealth 2.0: Experiences, Possibilities, and Perspectives. JMIR Mhealth Uhealth.

[28] Tomlinson M, Rotheram-Borus MJ, Swartz L, Tsai AC (2013). Scaling up mHealth: where is the evidence?. PLoS Med.

[29] Seiler A, Klaas V, Tröster G, Fagundes CP (2017). eHealth and mHealth interventions in the treatment of fatigued cancer survivors: A systematic review and meta-analysis. Psychooncology.

[30] Winslow BD, Chadderdon GL, Dechmerowski SJ, Jones DL, Kalkstein S, Greene JL, Gehrman P (2016). Development and Clinical Evaluation of an mHealth Application for Stress Management. Front Psychiatry.

[31] Bastien C, Vallières A, Morin C (2001). Validation of the Insomnia Severity Index as an outcome measure for insomnia research. Sleep medicine, Medline.

[32] Zimmerman BJ (2002). Becoming a Self-Regulated Learner: An Overview. Theory Into Practice.

[33] Kang S, Kang JM, Cho S, Ko K, Lee YJ, Lee H, Kim L, Winkelman JW (2017). Cognitive Behavioral Therapy Using a Mobile Application Synchronizable With Wearable Devices for Insomnia Treatment: A Pilot Study. J Clin Sleep Med.

[34] Bhat S, Pinto-Zipp G, Upadhyay H, Polos PG (2018). 'To sleep, perchance to tweet': in-bed electronic social media use and its associations with insomnia, daytime sleepiness, mood, and sleep duration in adults. Sleep Health.

[35] Sathyanarayana A, Joty S, Fernandez-Luque L, Ofli F, Srivastava J, Elmagarmid A, Arora T, Taheri S (2016). Sleep Quality Prediction From Wearable Data Using Deep Learning. JMIR Mhealth Uhealth.

